# Integrating mechanical cues in *in vitro* models of immune-related fibrotic diseases

**DOI:** 10.3389/fimmu.2026.1728712

**Published:** 2026-04-20

**Authors:** Chaoyang Song, Mengqi Zhu, Peiwen Li, Qiao Mao, Chongyuan Zhu, Ning Li, Yan Zhang, Yu Du, Mian Long

**Affiliations:** 1 Center for Biomechanics and Bioengineering, Key Laboratory of Microgravity and Beijing Key Laboratory of Engineered Construction and Mechanobiology, Institute of Mechanics, Chinese Academy of Sciences, Beijing, China; 2 School of Engineering Science, University of Chinese Academy of Sciences, Beijing, China; 3 School of Materials Science and Engineering, East China University of Science and Technology, Shanghai, China; 4 Key Laboratory for Biorheological Science and Technology of Ministry of Education, College of Bioengineering, Chongqing University, Chongqing, China

**Keywords:** extracellular matrix, fibrosis, immune cells, *in vitro* models, mechanical microenvironment, organoids, organ-on-a-chip

## Abstract

Fibrosis is a pathological process characterized by excessive deposition of extracellular matrix (ECM) and tissue stiffening, leading to organ failure and representing a common end-stage manifestation of numerous chronic diseases. The immune system plays a pivotal role in fibrosis progression, where various immune cells participate in the initiation and development of fibrosis by secreting various cytokines and regulating the balance between inflammation and repair. Mechanical signals such as ECM stiffness, fluid shear stress, and tissue viscoelasticity also significantly contribute to fibrotic progression. Alteration of mechanical microenvironment in fibrotic tissues not only influences fibroblast activation and ECM remodeling but also modulates immune cell recruitment, polarization, and function, thereby forming a pro-fibrotic positive feedback loop involving mechanical, immunological, and fibrotic responses. Conventional models, such as two-dimensional (2D) cell cultures and animal models, exhibit considerable limitations in recapitulating such complex cellular interactions. Recent advances in organoid and organ-on-a-chip (OoC) technologies provide powerful tools to better mimic *in vivo* multicellular crosstalk, mechanical microenvironment, and immune responses, facilitating the understanding of fibrotic mechanisms and screening of anti-fibrotic drugs. This review summarizes the pathological bases of immune-related fibrotic diseases, alterations in mechanical microenvironment, interactions between immune cells and fibrotic tissues, and highlights the application and prospects of organoid and OoC platforms in fibrosis research involving mechanical and immunological factors.

## 1 Interplay of immunity and mechanics in fibrosis

Fibrosis is a dysregulated wound healing response characterized by excessive deposition of extracellular matrix (ECM), leading to progressive tissue stiffening and ultimately organ failure (1, 2). Fibrotic diseases can be triggered by various factors, such as chronic inflammation, recurrent infections (e.g., chronic viral hepatitis), long-term exposure to toxic substances (e.g., silica or bleomycin), autoimmune abnormalities (e.g., systemic sclerosis), or metabolic disorders (e.g., non-alcoholic steatohepatitis). Regardless of the etiology, the end result is the replacement of functional parenchymal tissue with scar tissue (3, 4). Clinically, fibrosis-related diseases account for approximately 45% of all causes of death in industrialized nations, and there are currently no effective treatments. The economic burden they impose even exceeds that of many malignant tumors, making pathological mechanisms and treatment methods of fibrosis a persistent challenge (5).

Clinically, many diseases can cause organ fibrosis. For the heart, conditions such as post-myocardial infarction fibrosis (6), hypertensive heart disease (7), dilated cardiomyopathy (DCM) (8), and heart failure with preserved ejection fraction (HFpEF) (9) can all lead to cardiac fibrosis. Common pulmonary fibrosis diseases include idiopathic pulmonary fibrosis (IPF) (10), cystic fibrosis (CF) (11), interstitial lung disease (ILD) (12), silicosis (13), and COVID-19-induced pulmonary fibrosis (10). For the liver, common hepatic fibrosis diseases include liver cirrhosis, Metabolic dysfunction-associated steatotic liver disease (MASLD), alcoholic liver disease (ALD), primary sclerosing cholangitis (PSC), and viral hepatitis-induced fibrosis (14). In addition to the aforementioned fibrosis-related diseases, subcutaneous implantation can also induce tissue fibrosis. This process is also mediated by immune responses, involving both adaptive and innate immunity. Immune cells, such as macrophages, synergize with fibroblasts to create a pro-fibrotic microenvironment (15, 16).

Both immune responses and mechanical factors are critical in the regulation of fibrosis progression. On one hand, the immune system plays a crucial role in the progression of fibrotic diseases. During inflammation, persistent activation of immune cells results in abnormal tissue repair programs, leading to chronic inflammation and eventually fibrosis. Immune cells involved in this response, such as macrophages, T cells, and neutrophils, are recruited to the site of injury, where they secrete cytokines and growth factors that promote fibroblast activation and ECM synthesis (17). On the other hand, mechanical cues such as tissue stiffness, shear stress, and tension collectively regulate organ development, morphogenesis, and homeostasis in healthy tissues. In fibrotic organs, however, the mechanical environment of tissues undergoes various changes: both stiffness and viscoelasticity of ECM increase due to excessive deposition and cross-linking of ECM proteins such as collagen and fibronectin (2); hemodynamic forces are also disrupted in fibrotic tissues (18), as observed that portal hypertension in liver fibrosis causes elevated shear stress and hydraulic pressure in liver sinusoids, which could further promote the progress of fibrosis (19). Moreover, these mechanical changes also affect immune responses as immune cells are highly mechanically sensitive: macrophages can remodel their cytoskeleton within minutes of sensing a rigid matrix; full activation of T cells requires a specific range of matrix stiffness (2); and transendothelial migration of neutrophils is regulated by the stiffness of the subendothelial matrix (20). These clues illustrate the inseparable relationship between mechanical signals and immune responses in the progression of fibrosis, highlighting the importance of integrating mechanical cues when studying the role of immune cells in fibrosis.

Evidently, studying diseases or developing therapeutic treatments requires reliable *in vitro* models. Traditional *in vitro* models, such as cells seeded on petri dishes, are limited by the difficulty in achieving multicellular co-culture and replicating the mechanical microenvironment that immune cells experience *in vivo*. In recent years, *in vitro* models such as organoids, organ-on-a-chip (OoC), tunable hydrogels, and biological scaffolds have emerged with the capacity to precisely control mechanical factors while allowing co-culture of patient-derived immune, stromal, and parenchymal cells (21). This review summarizes recent advances in *in vitro* models applied in immune-related fibrotic diseases within a mechanical context: First, it outlines the current research status of immune-related fibrotic diseases; then, it compiles the major immune cell types involved in fibrosis and their mechanisms of interaction with the ECM; most importantly, it discusses the limitations of traditional *in vitro* models in fibrosis research, as well as the applications of emerging organoids and OoC in fibrosis research, and finally, it provides prospects for future fibrosis research.

## 2 Immune-related fibrotic diseases

**Figure 1:**
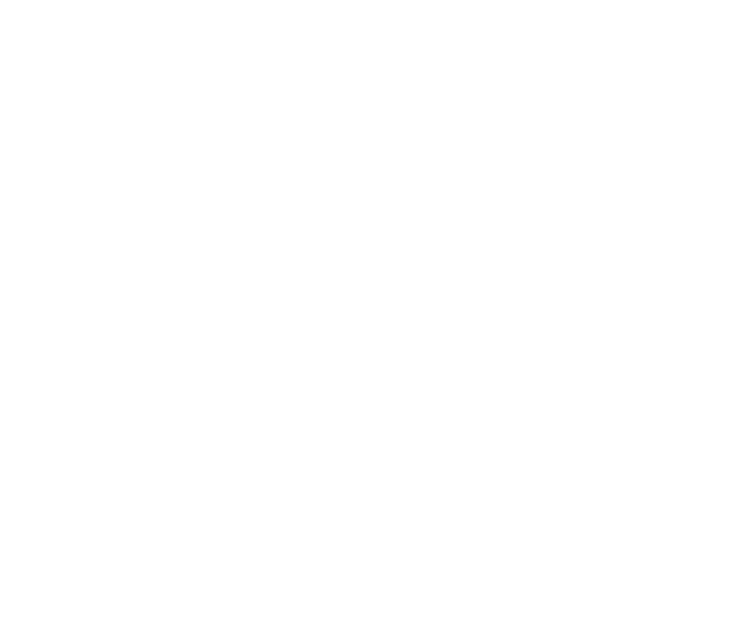
Roles of immune cells in fibrosis. Following tissue injury, immune cells infiltrate the damaged site in a temporally coordinated manner under the guidance of chemokines, subsequently undergoing phenotypic and functional adaptations. These cells release cytokines, along with pro-inflammatory/pro-fibrotic or anti-inflammatory/anti-fibrotic mediators, which act on fibroblasts and other immune cells. The persistent activation of fibroblasts further exacerbates ECM deposition and immune responses. This interplay modulates ECM deposition and contributes to the progression of tissue fibrosis.

As fibrosis progresses, immune cells are continuously recruited and activated (22, 23). Uncontrolled fibrosis often results in structural damage and progressive functional loss of organs such as heart, lungs, liver, and kidneys. Despite various triggers (pathogen infections, toxic injuries, or genetic factors), the development of fibrosis typically begins with an abnormal tissue repair response caused by cell senescence and tissue damage (24). Various immune cells are recruited to the injury site and activated to create a pro-fibrotic microenvironment, and the ECM is excessively deposited and cross-linked into a rigid network to further exacerbates tissue hypoxia and cellular dysfunction. These two aspects promote each other, forming a positive feedback loop that drives the progression of fibrosis (**Figure 1**).

### 2.1 ECM deposition and remodeling

The core pathological feature of fibrosis is the excessive ECM deposition and structural remodeling. In the physiological state, the synthesis and degradation of ECM such as collagen, fibronectin, and laminin from resident fibroblasts are dynamically balanced by matrix metalloproteinases (MMPs) and tissue inhibitors of metalloproteinases (TIMPs). However, during the process of fibrosis, this delicately maintained homeostasis is severely disrupted (**Figure 1**).

First, ECM synthesis is significantly increased during fibrosis (25). The key pro-fibrotic factor, transforming growth factor-β (TGF-β), promotes fibroblast activation, proliferation, and ECM synthesis by activating both classical Smad signaling pathway and non-classical pathways such as PI3K/AKT, MAPK (ERK), and JAK/STAT, thereby exacerbating the fibrotic process (26, 27). Furthermore, various pro-fibrotic cytokines, including platelet-derived growth factor (PDGF), connective tissue growth factor (CTGF), and interleukin (IL), amplify the pro-fibrotic effects either independently or synergistically with TGF-β (28–30). Meanwhile, ECM degradation is inhibited. TGF-β upregulates the expression of TIMPs, inhibits the proteolytic activity of MMPs. Overexpression of plasminogen activator inhibitor-1 (PAI-1) impedes the conversion of plasminogen to active plasmin, blocking the activation of MMP precursors and further limiting the capacity for ECM degradation (31).

Second, the composition and structure of ECM undergo pathological changes in fibrotic tissue. As fibrosis progresses, stiffer Col-I gradually replaces Col-III as the dominant component of ECM. Changes in basement membrane components and distribution of interstitial collagen disrupt tissue structure, destroy the original cell niches, and affect cell migration and phenotypic transformation. Excessive accumulation of fibronectin subtypes (such as FN-EDA) can continuously activate TGF-β via integrins to form a pro-fibrotic positive feedback loop. The increased Col-I fibers in ECM undergo covalent cross-linking catalyzed by lysyl oxidase (LOX), significantly enhance the ECM’s stiffness and stability and become more resistant to degradation (32). Excessive ECM deposition and structural remodeling also alter tissue mechanical microenvironment to drive the progression of fibrosis.

### 2.2 Pivotal roles of immune cells in the pathogenesis of fibrotic diseases

Organ fibrosis is a dynamic repair response under close regulation by immune cells. In this process, innate immune cells like neutrophils, macrophages, and NK cells first respond to tissue damage. Later, adaptive immune cells such as dendritic cells, T cells, and B cells participate in tissue remodeling (**Figure 1**).

Neutrophils, as critical innate immune effector cells, drive tissue and organ fibrosis through multiple mechanisms, including the release of pro-inflammatory and pro-fibrotic mediators, facilitation of fibroblast-to-myofibroblast transition, and formation of neutrophil extracellular traps (NETs). These processes exhibit organ-specific variations due to differences in injury signals and effector molecules (33). In cardiac injury, neutrophils promote collagen production and ECM deposition by releasing factors such as MMPs, IL-1β, TGF-β, and NOX4 (34). In liver, NETs activate hepatic stellate cells (HSCs) via binding to the NLRP3 inflammasome, leading to expression of α-SMA and Col-I (COL1A1) and subsequent IL-1β release, thereby exacerbating liver fibrosis (35, 36). In pulmonary tissue, NETs enhance fibroblast proliferation and differentiation into myofibroblasts via TLR9–miR-7–Smad2 pathway, contributing to interstitial lung disease and pulmonary fibrosis (37).

Macrophages are core components of innate immunity, which undergo phenotypic and functional changes responding to the injured tissue microenvironment, and generally exhibit the role of “promoting fibrosis in the early stage and promoting repair in the late stage” during the process of injury repair in different organs. During the initial stage of tissue injury, specific macrophage subsets, such as monocyte-derived macrophages, secrete various effector molecules including transforming growth factor-beta 1 (TGF-β1) and PDGF. These effector molecules drive fibrosis through the following mechanisms: (1) mediating fibroblast proliferation, migration, and transdifferentiation into myofibroblasts, thereby enhancing ECM deposition and tissue remodeling; (2) upregulating TIMPs to suppress MMP activity and promote collagen synthesis and ECM deposition; and (3) exerting synergistic actions with other immune cells. During the regression phase of tissue injury, distinct macrophage subsets such as tissue-resident macrophages and reprogrammed M2-polarized macrophages exert anti-fibrotic effects through the following pathways: (1) secreting proteolytic enzymes including MMPs and cathepsins to degrade ECM components; (2) clearing ECM components *via* integrin-mediated phagocytosis followed by lysosomal degradation; (3) secreting inhibitory factors such as interleukin-10 (IL-10) and arginase-1 (ARG1) to suppress fibroblast proliferation and activation; and (4) clearing apoptotic cells, such as apoptotic neutrophils, thereby inhibiting the progression of fibrosis.

In addition to the classical M1/M2 macrophage classification, recent advancements in single-cell RNA sequencing technology have identified macrophage subsets that play a key role in fibrosis progression, termed scar-associated macrophages (SAMs) (38). SAMs were first discovered in the liver and originate from circulating monocytes (39). These cells express at least five specific markers: TREM2, CD9, SPP1, GPNMB, and FABP5 (40). Although TREM2 has anti-fibrotic functions, the overall role of SAMs is pro-fibrotic. They can directly act on fibroblasts to promote various fibrotic phenotypes. Studies have shown that SAMs exert pro-fibrotic effects in multiple organs, including the heart, liver, and lungs (39).

Natural killer (NK) cells play a crucial anti-fibrotic role, primarily through direct cytotoxic targeting of activated fibroblasts. Specifically, in the myocarditis model, NK cells modulate eosinophil activity, thereby attenuating cardiac fibrosis (41). In chronic diseases, however, the anti-fibrotic capacity of NK cells is impaired due to the disruption of their adhesion and cytotoxicity (42, 43). Thus, the efficacy of NK cell-mediated anti-fibrotic responses is highly dependent on organ-specific contexts and disease stages.

Dendritic cells (DCs) are important contributors to fibrotic diseases. Due to their functional heterogeneity, different DC subpopulations play different and often opposite roles in the organ. Plasmacytoid DCs (pDCs) promote lung fibroblast activation and collagen deposition through the secretion of CXCL4 and IFN-α (44, 45). In contrast, certain DC subsets exert anti-fibrotic effects, for example, Flt3L-amplified CD11b^pos^ DCs are able to inhibit HSC activation, inhibit TGF-β/Smad signaling, and promote anti-inflammatory responses by inhibiting cytotoxic T cell function and promoting regulatory T (Treg) amplification (46–49).

B cells contribute to fibrosis through interactions with other cell types, primarily by promoting ECM production and fibroblast-to-myofibroblast differentiation via direct contact or secretion of profibrotic factors such as IL-6 and TGF-β (50). They also amplify inflammatory responses by driving Th17 cell differentiation and macrophage polarization, thereby indirectly exacerbating fibrotic processes (50, 51). Conversely, certain B cell subsets exhibit protective functions. Regulatory B cells (Bregs) produce IL-10, which has been shown to attenuate fibrosis in both liver and lungs, highlighting their role in mitigating fibrotic progression (52–54).

CD4^+^ T cells differentiate into various helper T (Th) and Treg subsets under cytokine influence, each playing distinct roles in fibrosis. Th1 cells exert anti-fibrotic effects by secreting IFN-γ, which inhibits fibroblast proliferation, collagen synthesis, and promotes myofibroblast apoptosis (55–57). In contrast, Th2, Th9, and Th17 subsets drive fibrosis through cytokines such as IL-4, IL-9, IL-13, IL-17, and IL-31, which stimulate ECM deposition, upregulate pro-fibrotic factors, and skew Th0 cells toward a Th2 phenotype, exacerbating fibrotic imbalance—particularly in pulmonary settings (57–61). Tregs demonstrate context-dependent roles: early in pulmonary fibrosis, they promote diseases via TGF-β1-mediated fibroblast activation, while they suppress inflammation and collagen accumulation at a later stage (62, 63). Meanwhile, CD8^+^ T cells exhibit dual roles in fibrosis. They promote diseases via chemotaxis to organs such as lung, liver, and heart, where they enhance cytotoxicity, inflammation, and fibroblast activation (64–67). Conversely, specific subsets, such as CD8^+^ tissue-resident memory T cells (Trm) in MASLD, ameliorate fibrosis through CCR5-dependent recruitment and induction of activated HSC apoptosis (68).

### 2.3 Effects of fibrotic microenvironment on immune cells

The fibrotic microenvironment also reciprocally shapes immune cell behaviors as immune cells play critical roles in the progression of tissue and organ fibrosis. Abundant chemokines are secreted by activated fibroblasts and damaged tissue cells to form a chemokine network within the fibrotic microenvironment that orchestrates immune cell recruitment, as exemplified by CXCL1/8/12 for neutrophils recruitment (69), CCL2 for monocytes that differentiate into macrophages (70), CXCL9/10 for Th1 cells (71), and CCL18 for CD8^+^ T cells (64). After recruitment, phenotype and function of immune cells can be modulated by biochemical and/or mechanical signals of fibrotic microenvironment. Biochemical signals (such as hypoxia, chemokines, and cytokines) have been well understood (72–75). Besides, changes in mechanical properties of fibrotic tissues (ECM stiffness and viscoelasticity) are also noted to influence immune cell function through mechanotransduction pathways, ultimately affecting the progression of various diseases (76). Mechanical forces are known to regulate multiple key responses of CD8^+^ T cells, such as migration, antigen presenting, and cytotoxicity (77). Thus, the fibrotic microenvironment remodels immune responses by modulating immune cell recruitment, phenotypic reprogramming, and functional alterations.

### 2.4 Cellular senescence drives fibrosis

Cellular senescence is a state of cell-cycle arrest and is now recognized as a significant driver of fibrotic diseases. Senescent cells secrete various substances through the senescence-associated secretory phenotype (SASP) to promote inflammation and fibrosis development (78, 79). Existing evidence indicates that fibrosis alters the stiffness of the ECM, thereby promoting cellular senescence. Using fibrotic substrates (with higher stiffness) is able to promote collagen secretion by human primary lung fibroblasts (IMR-90) and induces cellular senescence and a secretory phenotype (80). Furthermore, cellular senescence can activate the immune system through SASP secretion of inflammatory factors and chemokines (such as M-CSF, CCL2, and CXCL10), recruiting immune cells like macrophages, neutrophils, and T cells (81). Controlled SASP can promote wound healing and clearance of senescent cells *via* immune cells, whereas chronic SASP leads to prolonged inflammation, exacerbating fibrosis progression (82). Additionally, alongside immune system aging, the weakened proliferative capacity of immune cells and their decreased sensitivity to stimuli further aggravate pulmonary fibrosis. The senescence of immune cells, such as macrophages, can also lead to pro-fibrotic outcomes (83). Importantly, senescence and fibrosis form a bidirectional vicious cycle. TGF-β signaling, a major regulator of fibrosis, both induces cellular senescence and is upregulated by SASP factors from senescent cells. For example, senescent alveolar epithelial cells release TGF-β, activating fibroblasts and promoting myofibroblast differentiation, while senescent fibroblasts secrete SASP factors that perpetuate epithelial injury (84).

## 3 Mechanical cues in fibrotic diseases undergo significant alterations

### 3.1 Tissue stiffness​

**Figure 2:**
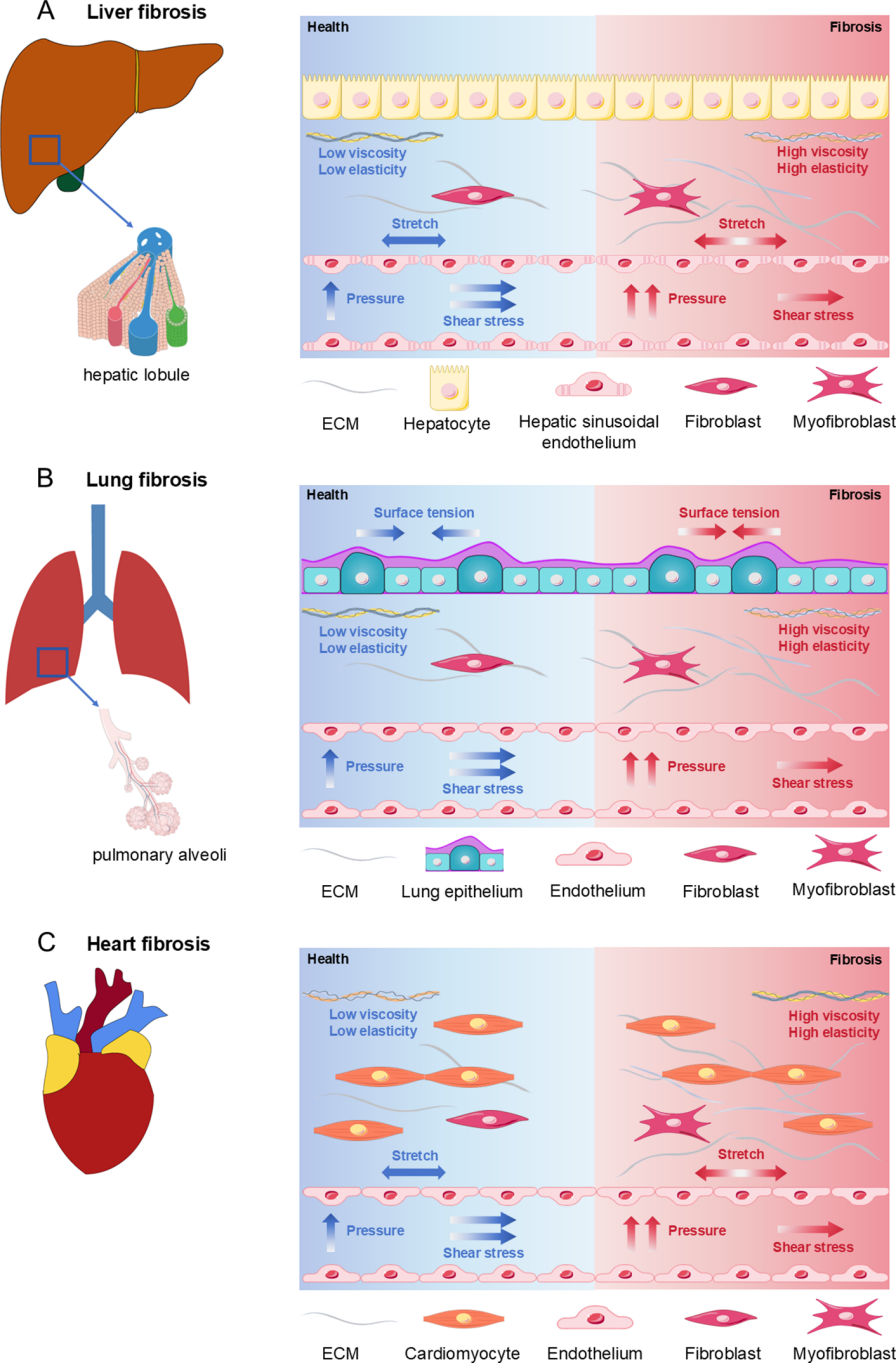
Schematic of mechano-microenvironmental alterations in liver, lung and heart fibrosis.​​ **(A)** Pathogenic stimuli activate HSCs, driving their transdifferentiation into myofibroblasts. This activation is accompanied by robust synthesis and secretion of ECM components, resulting in a marked increase in substrate stiffness. Concomitant hemodynamic perturbations include elevated intravascular pressure, enhanced cyclic circumferential wall strain, and disturbed low-magnitude shear stress within vascular lumen. Concurrently, the tissue exhibits viscoelastic reprogramming, manifesting as a transition toward a solid-like mechanical phenotype. **(B)** Excessive deposition of ECM contributes to increased tissue stiffness and altered viscoelastic properties, characterized by a shift toward a solid-like phenotype. This modified mechanical landscape further amplifies surface tension exerted on the pulmonary epithelium — including both airway and alveolar epithelial cells — as well as intravascular fluid shear stress and hydrostatic pressure. These aberrant mechanical cues perpetuate fibroblast activation and promote further ECM secretion, establishing a pathologic positive-feedback loop that exacerbates fibrotic progression. **(C)** Accumulation of ECM leads to increased myocardial stiffness and viscoelastic reprogramming, evidenced by a pronounced elevation in both elastic (storage modulus) and viscous (loss modulus) components, with a disproportionately dominant increase in viscous dissipation. Abnormal cardiac contractility and relaxation result in excessive vascular wall strain, elevated blood pressure, and reduced intravascular shear stress. These aberrant fluid and strain-related stimuli collectively activate cardiac fibroblasts, further promoting ECM remodeling, increased ventricular wall stress, elevated tissue stiffness, and ultimately impaired diastolic function.

Fibrotic diseases lead to a significant increase in the stiffness of affected organs (**Figure 2**), which has been consistently demonstrated across multiple tissue types. Stiffness is a critical mechanical property closely linked to physiological function. It is commonly characterized by the Young’s modulus, defined as Equation 1:

(1) $$E=\frac{\sigma }{\epsilon }$$

where *E* represents Young’s modulus, σ is stress, ϵ is strain.

In fibrotic organs, increased stiffness is observed at multiple scales using various measurement techniques. At the ECM and cellular levels, atomic force microscopy (AFM) nanoindentation is commonly employed to quantify the hardening of cells and ECM due to fibrosis (85–89). At tissue level, techniques such as AFM nanoindentation, rheometry, magnetic resonance elastography (MRE), shear wave elastography, and ultrasound elastography (UE) are used to assess the stiffness of fibrotic tissues. While AFM and rheometry can measure local tissue stiffness (88, 90–92), MRE, shear wave elastography, and UE provide bulk tissue stiffness measurements and can be performed *in vivo* (93, 94). Additionally, Brillouin microscopy is another tool used to quantify tissue stiffness, even though it has not yet been widely applied in studies of fibrotic organs. Increased stiffness not only serves as an indicator of fibrosis severity but also impairs normal physiological function—for example, elevated lung stiffness in pulmonary fibrosis hinders lung expansion and compromises respiration. The changes in stiffness of different organs after fibrosis are shown in **Table 1**.

**Table 1:** Summaries of mechanical parameters of various tissues.

Organ	Level	Method	Species	Normal organ stiffness (kPa)	Fibrotic organ stiffness (kPa)	References
Liver	Tissue	AFM	Mouse	0.15	1-6	(90)
		MRE	Human	2.2	5.8	(93)
		UE	Human	2.5	7.1-12.5	(90)
Heart	Tissue	AFM	Mouse	18	55	(91)
	ECM	AFM	Mouse	30.2-74.5	81.4	(85)
	Cell	AFM	Mouse	0.7	1.4	(86)
Lung	Tissue	Rheometer	Mouse	1.87	2.92	(92)
		AFM	Human	1.96	16.52	(88)
	ECM	AFM	Human	1.606	7.34	(88)
	Cell	AFM	Human	0.87	1.74	(89)

### 3.2 Hemodynamics of blood flow

Fibrotic diseases significantly alter blood flow within affected organs (**Figure 2**). Blood flow plays a vital physiological role by supplying nutrients and oxygen and exerting various mechanical forces on cells, such as fluid shear stress and blood pressure. Thus, fibrosis-induced changes in blood flow can profoundly affect nutrient and oxygen delivery as well as local mechanical microenvironment. Blood flow in vessels is described by Poiseuille’s law (Equations 2, 3) (95):

(2) $$Q=\frac{\pi r^{4}\text{Δ}P}{8\eta L}$$

(3) $$R=\frac{8\eta L}{\pi r^{4}}$$

where Q represents flow rate, r is vessel radius, Δ*P* is the pressure gradient along the vessel, L is vessel length, and R denotes fluid resistance. Measurements of flow rate, radius, and other parameters can be used to quantify the impact of fibrosis on organ perfusion. Hemodynamic consequences of fibrosis and optimal techniques for their measurement vary across organs owing to fundamental differences in vascular architecture, physiology, and other related factors. Current techniques for studying liver blood flow include angiography, Doppler ultrasound, and 4D flow MRI (96–101). In the heart, changes in blood flow are primarily assessed using techniques such as histology with section staining and angiography (102–105). Common methods for evaluating blood flow in fibrotic lungs include histopathological staining and phase-contrast MRI (106–110).

Specifically, the liver possesses a unique dual blood supply, with hepatic artery providing approximately 25% of oxygen-rich blood and portal vein supplying about 75% of nutrient-rich blood. Fibrosis is found to severely impair hepatic blood perfusion: in healthy individuals, the mean cross-sectional area of right portal vein is 72.6 mm² with a mean net flow rate of 5.02 mL/s, while, in severe cirrhosis patients, the values decrease to 41.7 mm² and 2.94 mL/s, respectively (98). The heart relies primarily on the coronary arteries for its blood supply, and cardiac fibrosis causes a median reduction of 27% in coronary microvascular density, further exacerbating myocardial ischemia and fibrosis (103). In the lungs, the pulmonary artery and pulmonary vein serve as the main vessels of pulmonary circulation, branching extensively into alveolar capillary networks to support nutrient delivery and gas exchange. In pulmonary fibrosis patients, over two-thirds yield at least moderate luminal narrowing in small pulmonary veins and venules, 65% exhibit occlusion of small pulmonary veins, and 73% develop pulmonary hypertension, indicating severe impairment of pulmonary blood flow due to fibrosis (108).

### 3.3 Viscoelasticity​

Fibrosis markedly alters the viscoelastic properties of organs (**Figure 2**). Viscoelasticity is an essential mechanical characteristic that reflects the dynamic behavior of tissues under load. It is typically characterized by the storage modulus *E*′ (representing the elastic component), the loss modulus *E*′′ (representing the viscous component), and the loss tangent tan*δ* = *E*′′/*E*′ that indicates the ratio of viscous to elastic behavior. The primary method for investigating viscoelasticity is AFM-based microrheology, which has been applied in examining the effects of fibrosis on the liver, heart, and lungs. For example, applying low-amplitude oscillatory forces at different frequencies enables one to define the viscoelasticity of the sample (85, 111–117).

Fibrosis commonly leads to increased elasticity and increased viscosity in organs (**Figure 2**). Mouse fibrotic liver tissue has a total stiffness of 1662.17 Pa and a cytoplasmic viscosity η of 59.00 Pa·s, compared to a normal liver with a total stiffness of 649.95 Pa and an η of 44.65 Pa·s (111). In mouse heart, under a loading frequency of 11.45 Hz (near the physiological heart rate), the elastic modulus (*G*′) of cardiac fibrotic areas is approximately 46 kPa, which is higher than that of healthy regions (10–33 kPa). At the same frequency, the loss tangent of fibrotic areas (tan*δ* = 0.53) is about twice that of healthy tissue (tan*δ* = 0.25), indicating markedly increased viscous energy loss and reduced mechanical efficiency in pumping blood (85). Similarly, in mouse lungs at a loading frequency of 0.01 Hz, fibrotic tissue has a storage modulus of 6.40 kPa and a loss modulus of approximately 0.5 kPa, whereas normal lung tissue has a storage modulus of 2.12 kPa and a loss modulus of about 0.2 kPa (116).

## 4 Mechanical cues modulate cell behaviors and fibrosis progression

Tissue-specific mechanical microenvironment undergoes dynamic changes as fibrosis progresses, involving alterations in matrix stiffness and viscoelasticity, interstitial fluid pressure and shear stress, and tensile forces. These mechanical changes primarily result from excessive ECM deposition, remodeling, and contractile activity of myofibroblasts. Notably, these mechanical alterations are not only consequences of fibrosis development but also play a significant role in regulating fibrosis. They jointly influence cell behaviors and fate within tissues alongside biochemical factors.

### 4.1 Fibroblasts

Activation of fibroblasts and their secretion of ECM are central to fibrosis progression. Resting fibroblasts, typically located around blood vessels and in the interstitium, are in a state of cell cycle arrest with low expression of α-SMA and ECM genes. When tissue damage occurs, damage-associated molecular patterns (DAMPs) such as ROS, HMGB1, IL-1β, and TNF-α are released locally. These molecules activate signaling pathways including TGF-β, TLR4/NF-κB, and PDGFR autophosphorylation cascades. This induces resting fibroblasts to upregulate the expression of molecules such as α-SMA, fibronectin, and PDGFRβ, acquiring the ability to proliferate and migrate (118). Subsequently, TGF-β1, via Smad2/3/4 complex and in collaboration with transcriptional coactivators YAP/TAZ, activates the transcription of downstream target genes, promoting ECM synthesis and deposition. Meanwhile, YAP/TAZ within the nucleus bind to TEAD transcription factors, driving the expression of pro-fibrotic genes such as CTGF, SPP1, and TGFβ, and promoting the transformation of fibroblasts into myofibroblasts (119).

During this process, tissue mechanical microenvironment plays a role in regulation through multiple mechanisms. First, the typical mechanoreceptor protein, integrin, on the cell membrane connects ECM to cytoskeleton, forming a cellular mechanosignaling network (120). Excessive deposition and cross-linking of ECM increase matrix stiffness, promoting the clustering of integrins on cell membrane. This activates focal adhesion FAK and downstream RhoA/ROCK pathways, enhancing contractile forces of myosin II and forming stress fibers (121–123). The viscoelasticity of healthy tissues can reduce fibroblast spreading and focal adhesion, inhibiting fibroblast activation. Abnormalities in the ratio of ECM components and cross-linking lead to increased matrix stiffness, and this alteration inhibits the release of cytoskeletal tension, maintaining YAP/TAZ activity and a pro-fibrotic transcriptional program (124, 125). In addition to this classical pattern of integrin adhesion-dependent mechanotransduction, mechanical microenvironment dynamically regulates nuclear pore morphology, where nuclear pore opening promotes the nuclear import and retention of YAP/TAZ (126). This binding to transcription factors such as TEAD continuously activates the expression of pro-fibrotic genes (127). Second, mechanically sensitive ion channel Piezo1 responds to various mechanical changes in cellular microenvironment, such as stretching forces, fluid shear stress, and pressure. Stretching promotes fibroblast activation and ECM deposition through Piezo1-mediated ERK phosphorylation and Ca^2+^ influx (128, 129). Furthermore, inflammatory conditions with supraphysiological interstitial flow activate TGFβ1, promoting fibroblast activation, oriented alignment, and matrix remodeling (130). Notably, during fibrosis progression, myofibroblasts transmit forces to neighboring fibroblasts by contracting collagen fibers, activating their transformation into myofibroblasts (17).

### 4.2 Other cells

In addition to fibroblasts, several other types of cells sense biomechanical and biochemical signals to participate in fibrosis. Stretch forces cause senescence in alveolar epithelial cells, promoting fibroblast activation and epithelial-mesenchymal transition (EMT) in pulmonary epithelial cells via paracrine signaling (131, 132). A vicious cycle of myofibroblast contraction and matrix stiffening accelerates pulmonary fibrosis. Alveolar type II epithelial cells on stiff substrates inhibit E-cadherin expression and upregulate Snail/Twist transcription factors through the integrin-FAK-RhoA/ROCK pathway and YAP nuclear translocation, driving EMT. They transform into α-SMA-positive myofibroblasts and secrete collagen I/III, fibronectin, TGF-β, and CTGF (133, 134).

Endothelial cells are highly sensitive to mechanical signals. Increased tissue stiffness and altered blood flow pressure and shear stress disrupt the VE-cadherin/β-catenin complex, increasing vascular permeability and promoting inflammatory exudation (135). Meanwhile, Piezo1 channel activation triggers the calcineurin-NFAT and integrin-FAK-MRTF pathways, inducing endothelial-mesenchymal transition (EndMT). This directly transforms endothelial cells into collagen-secreting mesenchymal cells, exacerbating microvascular rarefaction (136). Vascular pericytes protect vascular integrity in healthy tissues, while during tissue fibrosis, increased matrix stiffness activates the YAP signaling pathway, inducing the transition of pericytes into fibroblasts and enhancing their invasiveness (137).

During fibrosis, the tissue mechanical microenvironment changes. These changes include myofibroblast contraction, matrix remodeling, local hemodynamic alterations, and inflammation-induced increases in interstitial fluid pressure. They modify the niche of tissue-resident cells. These cells sense and transmit mechanical signals. Then, via downstream signaling pathways, the cells exhibit behaviors like migration, transformation, abnormal secretion, and apoptosis resistance, which regulate fibrosis progression. Studies have revealed that stage-specific cellular fate and behavior emerge during the progression of fibrosis, which are dynamically shaped by the extracellular mechanical microenvironment. This connection suggests the potential of biomechanics-based therapeutic strategies. However, the difficulty in quantitatively capturing the dynamically shifting mechanical milieu *in vivo* presents a major obstacle. This reality underscores the urgent need to develop more sophisticated biomechanical research models to facilitate deeper study.

## 5 Current *in vitro* models

### 5.1 Traditional fibrosis models

Development of effective antifibrotic therapies relies on robust disease models. Traditional fibrosis models, such as animal models, two-dimensional (2D) cell culture, and precision-cut tissue slices, have been used for many years and offer numerous advantages. For example, mouse models are most widely used, due to their sharing over 95% genetic homology with humans (118), editable genomes, rapid breeding, and low cost. Fibrosis can be induced chemically (*e.g.*, ethanol, carbon tetrachloride (CCl_4_), thioacetamide (TAA), dimethylnitrosamine (DMN), diethylnitrosamine (DEN), or other hepatotoxins), *via* dietary manipulation (methionine-choline-deficient diet (MCD), high-fat diet (HFD), Western diet (WD), choline-deficient diet, or L-amino acid-defined diet (CDAA)), surgically (*e.g.*, bile duct ligation, BDL) or *via* immune injury (4, 14, 138). 2D culture, using primary cells or cell lines (*e.g.*, LX-2, A549) (14, 139) are low-cost and tractable. Precision-cut tissue slices preserve native three-dimensional (3D) architecture, physiological ECM, and multicellular interactions of the tissue. These slices are sourced from healthy or diseased human or animal tissues and stimulated with pro-fibrotic agents (*e.g.*, TGF-β, bleomycin) (140, 141). Traditional models also exhibit evident limitations. For example, mouse models only partially recapitulate human diseases, and interspecies differences contribute mechanistically to the failure of numerous drug candidates in clinical trials. There are differences in the sensitivity of immune cells and hepatic stellate cells from humans and mice to fibrotic factors and lipopolysaccharide (LPS) (4). 2D culture fails to emulate *in vivo* 3D architecture, multicellular crosstalk, and biomechanical cues. Human precision-cut tissue slices are often limited to end-stage diseased tissues, and this precision-cut tissue slices platform suffers from low throughput, short viability, and batch-to-batch variation.

### 5.2 Organoids and organ-on-a-chip

#### 5.2.1 Organoids

Organoids are 3D self-organizing structures derived from stem cells or patient tissues, cultured in ECM-mimetic matrices (*e.g.*, Matrigel). They replicate key aspects of tissue architecture and allow personalized disease modeling and high-throughput drug screening.

**Figure 3:**
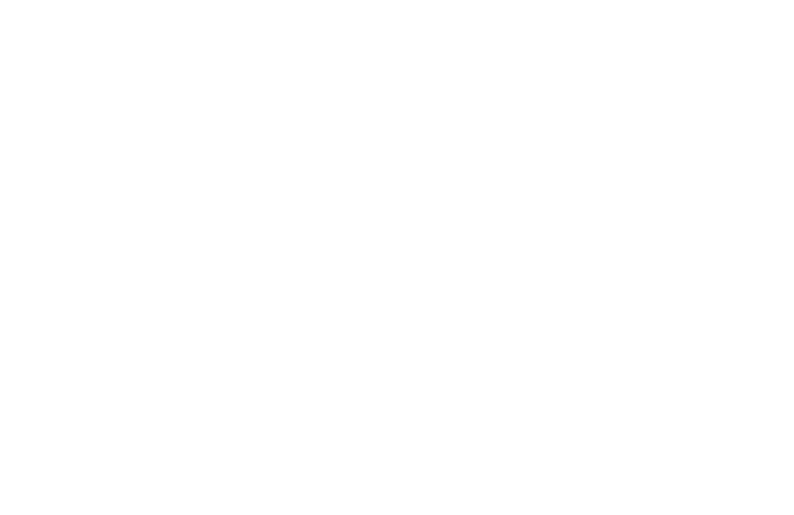
Immune processes involved in the progression of fibrosis in the lung, liver, and heart, along with organoid and organ-on-a-chip platforms used for fibrosis research. For pulmonary fibrosis, the cells and molecules involved in the immune response at the alveolar-capillary interface are introduced. Patient-derived alveolar epithelial progenitors can be directly cultured to generate organoids that closely resemble fibrotic tissue in terms of cellular composition and structure. Lung-on-a-chip platforms can simulate the mechanical microenvironment of the lung, such as mechanical stretch due to breathing and fluid shear stress from airflow and blood flow, and can also be integrated with immune cells to study immune responses. For liver fibrosis, the cells and molecules involved in the fibrotic immune response within the liver lobule unit are described. Hydrogels with tunable stiffness can be used to mimic the fibrotic microenvironment for culturing liver organoids, revealing that increased matrix stiffness promotes fibrogenesis. Additionally, liver organoids can be coupled with vascular and immune components and serve as advanced tools for drug screening. Liver-on-a-chip platforms enable co-culture of multiple hepatic cell types, including primary human hepatocytes, Kupffer cells, liver sinusoidal endothelial cells, and hepatic stellate cells, thereby recapitulating the *in vivo* liver microenvironment. Exposure of such chips to lipotoxic conditions allows for the study of non-alcoholic steatohepatitis. For cardiac fibrosis, the immune cells and molecules involved in fibrotic cardiac tissue are outlined. Cardiac organoids can be induced to exhibit fibrotic phenotypes through physical or chemical injury. Furthermore, heart-on-a-chip platforms can incorporate electrophysiological functionalities, and microstructures such as micropillars and microrods can be employed to assess the mechanical properties of fibrotic tissue. Reproduced by the permission from the references (142, 147, 148, 152, 160, 162).

Lung organoids can accurately mimic pathological features of idiopathic pulmonary fibrosis and form cystic structures of honeycomb lung observed clinically, which is hard to achieve from animal models. Typically, a 3D organoid model is constructed using alveolar basal cells derived from IPF patients in Matrigel, which after 21 days of culture can self-assemble into organoids containing basal cells, ciliated cells, and secretory epithelial cells (such as goblet cells), and is very similar to the honeycomb cyst structures in the tissues of IPF patients (**Figure 3**). 40% of the organoids form polarized luminal structures similar to those *in vivo*, and these organoids can be used to detect the expression of IPF fibrotic marker MMP7 (142). Meanwhile, fibrotic organoids serve as a promising tool for drug screening. For example, fibrotic lung organoids are applied to test anti-fibrotic effects of pirfenidone and nintedanib, where pirfenidone significantly inhibits fibroblast proliferation but seems toxic to epithelial cell growth but nintedanib is safer (143).

Liver organoids can achieve multicellular co-culture, which is hard to accomplish with conventional 2D culture. For instance, a multicellular co-culture organoid system is established using adult stem cells, cholangiocytes, and mesenchymal cells. They first generate hepatocyte organoids with functional vascular network and then add cholangiocytes and portal fibroblasts for co-culture to recapitulate the structure of liver periportal region. These organoids can produce bile and drain it into bile ducts, also able to simulate cholestatic injury and biliary fibrosis features. Furthermore, immune cells can be incorporated into organoids for co-culture, as demonstrated by introducing Th17 cells into organoids and promoting fibrosis in PSC by miR-17a derived from Th17 cells (**Figure 3**) (144). Notably, organoids can also be subjected to mechanical stimuli. To study non-alcoholic fatty liver disease (NAFLD), engineered hydrogels and human induced pluripotent stem cells (hiPSCs) are used to construct a hepatic organoid. This hydrogel has tunable stiffness, which can simulate varied stiffness of fibrotic or healthy tissue. At high stiffness, hepatic lipid droplet accumulation increases, and the expression of liver fibrosis markers also increases. Moreover, inhibiting ROCK signaling pathway disrupts this synergistic effect (**Figure 3**) (145).

Cardiac organoids can achieve physiological contraction *in vitro*. A 3D cardiac organoid is constructed using neonatal rat primary ventricular cardiomyocytes combined with patterned hyaluronic acid (HA) substrates. The cardiomyocytes continuously contract, leading to gradual compression and detachment of entire cell layer, eventually spontaneously curling into a cylindrical 3D structure from the substrate, and achieving autonomous beating (146). Furthermore, by applying pathological stimuli, these organoids can be transformed into disease models, as observed by successfully simulating the cardiac fibrosis process following acute myocardial infarction (AMI) using a method combining ischemia-reperfusion (IR) injury and TGF-β1 stimulation (**Figure 3**) (147). Moreover, these organoids exhibit electrophysiological anomalies and contractile dysfunction, more closely resembling the pathological characteristics in the human body (147).

Despite their promise, organoids still face several remarkable limitations. Current technologies often yield organoids with insufficient maturity, limited physiological size, and reduced architectural complexity. Multicellular co-culture systems remain underdeveloped, frequently failing to emulate authentic cell-cell interactions *in vivo*. Moreover, organoids poorly recapitulate key physiological cues such as biomechanical forces, which are critical for realistic tissue development and disease modeling. Further challenges include limited reproducibility and a lack of standardized protocols, hindering broader application and validation.

#### 5.2.2 Organ-on-a-chip

OoC is a microengineered cell culture system that uses microfabrication to replicate key anatomical structures, tissue interfaces, and functional units of human organs—such as the alveolar-capillary barrier. These platforms enable precise multicellular co-culture under perfusion, allowing controlled delivery of biochemical and physical stimuli—including cyclic stretch, fluid shear stress, and compression—to recreate physiologically relevant microenvironments. As a result, OoC systems serve as powerful tools for real-time monitoring via integrated biosensors and for biomechanical studies. Beyond single-organ modeling, OoC technique also supports multi-organ co-culture, facilitating investigation of inter-organ crosstalk and systemic responses, making it an advanced platform for mechanistic studies in physiology and diseases, as well as for high-quality drug testing and screening.

Lung-on-a-chip: Alveolus-on-a-chip achieves the key functional interface of the tissue (the alveolar-capillary interface) and simulates physiological breathing (148). To replicate the features of pulmonary fibrosis in the chip, primary bronchial epithelial cells derived from CF patients are co-cultured with human pulmonary microvascular endothelial cells. Compared to chips cultured with healthy bronchial epithelial cells, the CF bronchial chip exhibits increased mucus secretion, elevated cilia density, and higher ciliary beating frequency, which is consistent with the pathological characteristics of CF patients. Additionally, enhanced secretion of IL-8 and increased migration of polymorphonuclear leukocytes are achieved (**Figure 3**) (149). Respiratory stretch also impacts the fibrotic phenotype. Equiaxial stretch (simulating respiratory strain) is applied to healthy and fibrotic lung ECM slices using an OoC. The stiffness of fibrotic ECM is significantly greater than that of healthy ECM in the absence of stretch, but the outcome is inverted when stretch is applied. This indicates that cyclic stretch during breathing may reduce the stiffness of fibrotic ECM, indirectly inhibiting the pro-fibrotic phenotype of fibroblasts (**Figure 3**) (21, 150).

Liver-on-a-chip: The ability to conveniently apply various external stimuli is a significant advantage of OoC systems, favoring the mimicry of multiple types of liver fibrosis diseases. To model alcoholic liver disease, a liver injury-on-a-chip is constructed by co-culturing hepatocytes and HSCs with biosensors specific for real-time TGF-β detection. Alcohol exposure activates stellate cells and initiates a pro-fibrotic feedback loop via TGF-β cross-talk between hepatocytes and HSCs, underscoring the value of multicellular models (151). To model MASLD, a multi-cellular MASLD chip is created by incorporating human hepatocytes, Kupffer cells, HSCs and endothelial cells. Under lipotoxic conditions, this system recapitulates MASLD hallmarks—pro-fibrotic marker elevation, HSC activation, and inflammation—and responds therapeutically to elafibranor, demonstrating its potential in drug testing applications (**Figure 3**) (152). In addition to chemical stimuli, mechanical stimuli can also be integrated into liver-on-a-chip systems. A vascularized bile duct-chip is developed using primary cholangiocytes from healthy and sclerosing cholangitis patients, indicating that shear stress aligns endothelial cells and supports vascular barrier formation, even though cholangiocytes are less responsive to flow (153, 154).

Heart-on-a-chip: OoC systems can integrate sensors for functional readouts, used to monitor the pathological features of fibrosis (electrophysiological abnormalities and contractile dysfunction). A hallmark of cardiac fibrosis is the decline in tissue contractility, which impairs pumping function and ultimately leads to heart failure. To quantify this dysfunction, OoC incorporates advanced force-sensing techniques, including Polydimethylsiloxane (PDMS) microcantilevers or microrods for both active and passive force measurements (**Figure 3**) (155), GelMA micropillars for strain analysis (156) and Biowire II platform using deflectable poly (octamethylene maleate (anhydride) citrate) (POMaC) wires to assess contractility and electrical coupling simultaneously (**Figure 3**) (157). Those non-invasive optical systems based on structural color hydrogels enable real-time quantification of contractile forces through spectral shifts, providing multifaceted functional readouts in fibrotic cardiac models (158). Additionally, the perfusable nature of OoC systems offers an excellent tool for studying the role of the immune system in fibrosis. When modeling SARS-CoV-2-induced myocarditis, a pro-inflammatory microenvironment is created by perfusing PBMCs, thus reducing contractility, elevating cytokine release, and causing mitochondrial damage—key events preceding fibrosis (159). Moreover, these systems can be further coupled with imaging and detection systems to achieve real-time *in situ* observation.

In fact, OoC still faces several challenges. It usually involves microfluidic and microfabrication technologies, which have a high entry barrier and are complex to operate. The 3D structures inside OoC are not self-formed and thus differ from *in vivo* conditions. Data obtained from OoC are difficult to analyze. OoC manufacturing mainly relies on manual fabrication, leading to the issues of stability, scalability, and standardization.

## 6 Discussion and future perspective

Severe fibrosis represents a common end-stage manifestation across numerous chronic diseases, culminating in organ failure and presenting persistent challenges in both mechanistic understanding and therapeutic intervention. While fibrosis is traditionally associated with persistent inflammation, recurrent injury, genetic predispositions, and metabolic dysregulation, it is increasingly clear that its pathogenesis extends beyond mere collagen deposition and fibroblast activation. The immune system is now recognized as a key contributor to fibrotic progression.

Conventional *in vitro* models provide foundational insights but suffer from critical limitations. In subcutaneous implantation models used for fibrosis research, rat and mouse models are utilized predominantly, which exhibit interspecies differences compared to humans. While the biochemical and mechanical environment of these subcutaneous implantation models is physiological, the complexity leads to difficulties in quantifying and decoupling, as well as a lack of high-throughput capability. Compared to *in vitro* models, subcutaneous implantation models possess a complete immune system. Furthermore, OoC can be engineered to incorporate implants to recapitulate the foreign body response (161).

**Figure 4:**
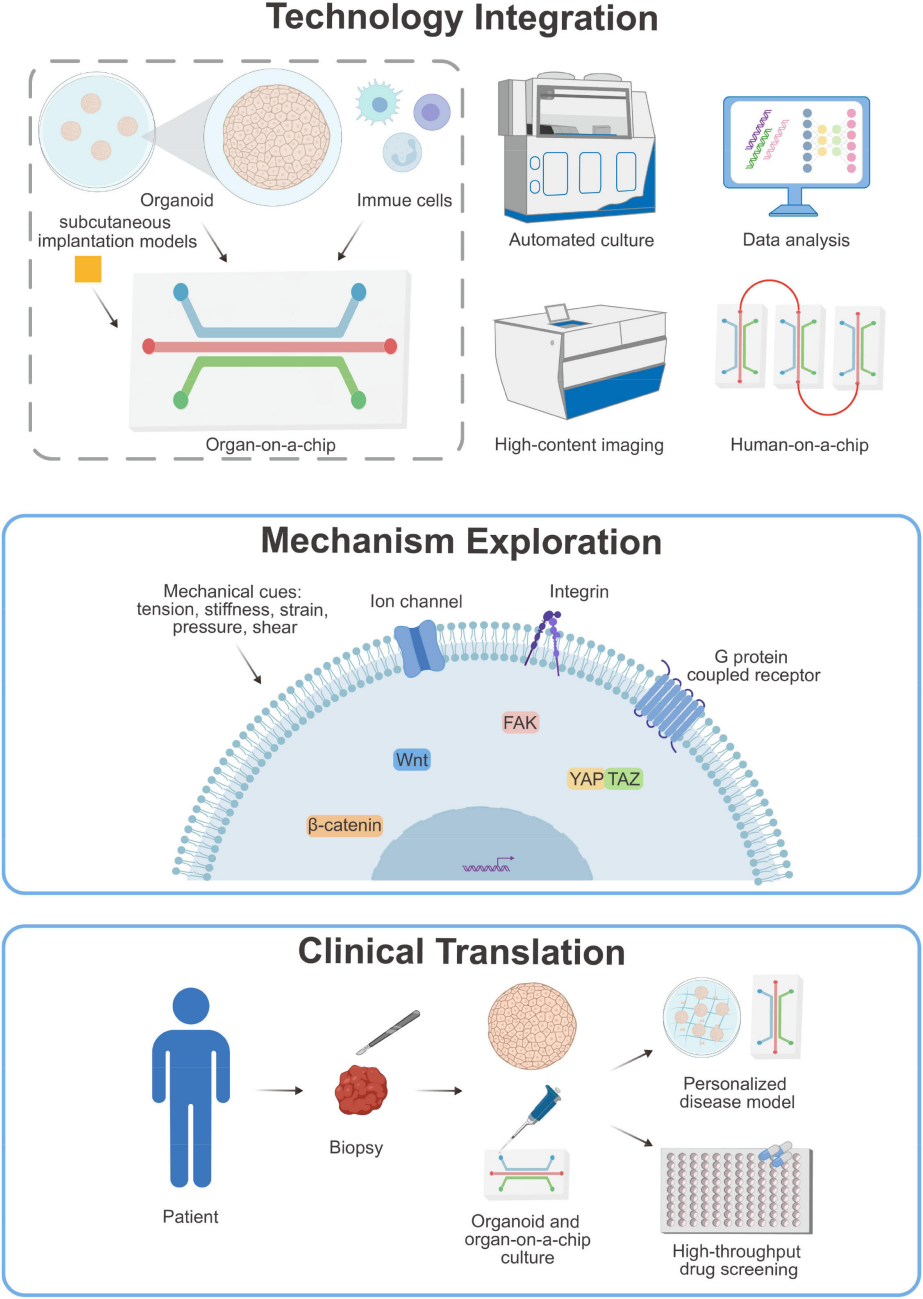
Future development of *in vitro* models relevant to fibrosis. Organoids-on-a-chip combine the advantages of both organoid and OoC technologies and can be integrated with *in vivo* implantation techniques. Future iterations should further incorporate a more complete immune system. Additionally, organoids-on-a-chip should be combined with automated equipment for culture, imaging, detection, and analysis. Mechanobiology-related molecules and pathways also represent a promising future direction and may emerge as novel therapeutic targets for fibrotic diseases. Organoid and OoC technologies offer the significant potential for personalized medicine by enabling the construction of patient-specific models derived directly from patient cells, which can be used to analyze the disease etiology and test more effective treatment strategies.

Recent advances in organoid and OoC systems offer promising avenues for overcoming these constraints. Fibrotic organoids can be constructed using patient-derived cells for better replication of human responses. OoC platforms enable multi-cellular co-culture and the application of biochemical and mechanical stimuli, allowing the construction of highly biomimetic tissue architectures and physiological microenvironments, with the capability to incorporate sensors for real-time monitoring. Furthermore, both of the techniques allow for the integration of immune cells. Nevertheless, current models still fall short of fully capturing the physiological complexity of fibrotic tissues. Organoids often lack perfusable vasculature and biomechanical inputs, while OoC may oversimplify tissue anatomy and cellular diversity. A synergistic approach—organoid-on-a-chip—aims to merge the biological fidelity of organoids with tunable microenvironment of microfluidic systems (**Figure 4**). Future iterations should strive to incorporate immune, neural, and vascular components to better mimic *in vivo* conditions (**Figure 4**). Multi-organ microphysiological systems (*e.g.*, “human-on-a-chip”) could further elucidate inter-organ crosstalk in systemic fibrotic diseases (**Figure 4**). Substantial hurdles remain in standardizing, scaling, and automating these platforms.

Integrating advanced technologies such as automated culture, high-content imaging, and machine learning-assisted data analysis into *in vitro* models is crucial for enabling large-scale, reproducible investigations into fibrotic mechanisms and drug responses. Notably, the incorporation of relevant mechanical cues remains rudimentary in most models. Future studies should incorporate multi-modal mechanical stimuli—such as pressure gradients, dynamic stiffness, and cyclic strain—to better emulate the pathophysiological microenvironment. The regulatory mechanisms of mechanotransduction (mechanosensitive ion channels (*e.g.*, PIEZO1), integrin-mediated signaling, and downstream pathways) in immune-fibrotic crosstalk warrant deeper investigation. For clinical translation, utilizing patient-derived cells in these engineered systems will also enhance personalized disease modeling and therapeutic screening (**Figure 4**).

In conclusion, the integration of mechanical and immune cues in next-generation *in vitro* models—through the convergence of biology, engineering, and immunology—holds great potential to unravel the complex mechanisms-driven tissue fibrosis. These advanced systems will not only deepen our fundamental understanding but also accelerate the development of effective anti-fibrotic therapies.
